# Enhancement of Fertilizer Efficiency Through Chinese Milk Vetch and Rice Straw Incorporation

**DOI:** 10.3390/plants14020246

**Published:** 2025-01-16

**Authors:** Tahir Shah, Adnan Anwar Khan, Yahya Mohammed Ali Aljerib, Muhammad Tariq, Donghui Li, Mingjian Geng, Yajun Gao, Qiang Zhu

**Affiliations:** 1 College of Resources and Environment, Huazhong Agricultural University, Wuhan 430070, China; tahirhzau@gmail.com (T.S.); y.jerep2024@gmail.com (Y.M.A.A.); lidh@webmail.hzau.edu.cn (D.L.); mjgeng@mail.hzau.edu.cn (M.G.); 2 College of Natural Resources and Environment, Northwest A&F University, Yangling 712100, China; adnan@nwafu.edu.cn (A.A.K.); m_tariq@nwafu.edu.cn (M.T.)

**Keywords:** Chinese milk vetch, rice straw, chemical fertilizer, fertilizer use efficiency, sustainability index

## Abstract

The incorporation of rice straw (RS) and Chinese milk vetch (CMV) with reduced chemical fertilizers (CFs) is a viable solution to reduce the dependency on CF. However, limited research has been conducted to investigate the impact of CMV and RS with reduced CF on rice production. A field trial was conducted from 2018 to 2021 with six treatments: CK (no fertilizer), F100 (100% NPK fertilizer (CF)), MSF100 (100% CF+CMV and RS incorporation), MSF80 (80% CF+CMV+RS), MSF60 (60% CF+CMV+RS), and MSF40 (40% CF+CMV+RS). The results revealed that compared with the F100, the MSF80 treatment maintained a significantly higher mean grain yield over the four years, with an increase of 5.8~24.5%. MSF80 treatment also improved nitrogen (N), phosphorus (P), and potassium (K) use efficiencies, sustainable yield index, and partial factor productivity. Soil organic matter (SOM), total nitrogen (TN), ammonium N (NH_4_^+^-N), nitrate N (NO_3_^−^-N), available phosphorus (AP), and available potassium (AK) contents were significantly enhanced under MSF80 across different growth stages in both 2020 and 2021 seasons over F100. Pearson correlation analysis revealed a strong positive correlation among SOM, TN, NH_4_^+^-N, AP, AK, and rice yield. Additionally, Partial Least Squares Path Modeling (PLS-PM) demonstrated significant relationships between organic amendments, soil nutrients, nutrient uptake, and yield. The above findings suggest that combining RS returning with CMV incorporation is a long-term sustainable strategy for maintaining soil health, and it could reduce fertilizer addition by 20% without prejudicing rice grain yield under a rice-green manure rotation system.

## 1. Introduction

Rice (*Oryza sativa* L.) is a fundamental food source for almost 50% of the global population, and the areas where it is grown account for 11% of the world’s cultivable land [1]. China accounts for 27.4% of the world’s average yearly rice production and consumption, making it the world’s most significant producer and consumer of rice crop [2]. The present agricultural strategy in China primarily emphasizes improving the yield per hectare and overall productivity to guarantee the stability and sustainability of rice production [3]. Rice grain yields have surged in recent decades, mainly due to the increased use of mineral fertilizers; however, excessive use of CF also coincided with a decrease in nutrient use efficiency, resulting in environmental consequences for soil, water, and air quality [4]. Deteriorating soil quality, such as acidification and structural damage due to high chemical fertilization, has already negatively impacted rice yield [5]. More grain yields with lower environmental impacts are thus a significant challenge for sustainable rice production [6]. In recent years, there has been a growing interest in substituting inorganic fertilizers with organic replacement, highlighting the need for further research to explore sustainable practices that improve rice production with minimal environmental impacts.

Green manure (GM) incorporation is a sustainable agricultural technique that reduces soil deterioration and increases crop yields while minimizing the reliance on CF [7]. According to the previous study, the application of GM doubled rice yields and a sustainable yield index and enhanced the soil’s physical-biological properties and nutrient cycling [8]. In addition, GM reduced the synthetic N input and enhanced the N use efficiency (NUE) [9]. Other studies have shown that GM effectively promotes carbon (C) sequestration and soil C distribution, further enhancing its role in sustainable farming systems [10]. Therefore, utilizing GM during winter fallow is an alternative for increasing rice production and reducing CF inputs.

CMV, as a GM in southern China, is one of the dominant GM crops in the rice cropping system. It fixed the N from the atmosphere, activated soil P and K, enhanced nutrient utilization, and released a considerable amount of nutrients after decomposition, mainly N, which is important for enhancing rice yield and nutrient uptake, as well as sustaining soil N pools [11,12]. The use of CMV alone as a GM is not always sufficient to significantly increase rice productivity and soil fertility. While studies have shown that incorporating CMV can improve SOM, N availability, and microbial activity, its effects on rice yield and long-term soil fertility are often limited unless combined with other practices or amendments. For instance, co-incorporation with RS or partial substitution of CF has been found to enhance nutrient availability and achieve higher rice yields compared to using CMV alone [13,14].

Co-incorporation of RS and leguminous GM is an effective soil fertility management strategy in southern China [15]. Previous research suggested that using GM with other organic materials can extend its favorable impact on soil nutrients and crop productivity. Combining GM with crop residues having a high C/N ratio yielded more benefits for succeeding crops than using only straw or GM [16]. Other studies have shown that returning crop residues increased SOM content, maintained soil productivity, and improved soil fertility [17]. RS has a high C/N ratio; thus, it decomposes slowly in soil and usually causes short-term immobilization of soil nutrients [18]. On the other hand, legume-based GM decomposes quickly due to its low C/N ratio. Research has shown that as much as 90% of the organic N contained in fresh GM residues can be converted to mineral form within a four-week period after being mixed into the soil [15]. Interestingly, it has been shown that combining leguminous GM with RS regulates the mixture additive’s C/N ratio, improving soil fertility and enhancing soil quality in rice-green manure cropping systems [9,19,20]. However, little is known about the ideal balance of CMV as a GM and RS when utilizing reduced CF in the rice cropping system. We hypothesize that integrating CMV and RS with reduced CFs will maintain rice yield and significantly improve soil health compared with traditional fertilizer application practices. Therefore, the current research aimed to develop a long-term rice cropping system based on reduced CF by incorporating GM and RS. This approach will provide a theoretical foundation for sustainable strategies for local farmers and similar cropping systems.

## 2. Results

### 2.1. Soil Properties

**Figure 1:**
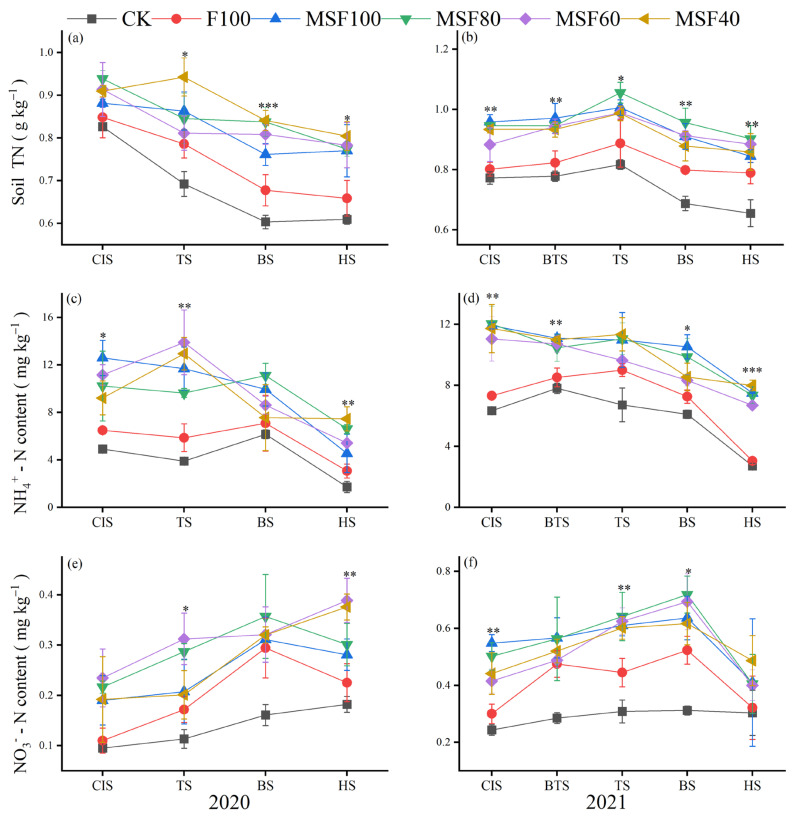
Soil total nitrogen (TN) (**a**,**b**), ammonium N content (NH_4_^+^ -N) (**c**,**d**), and nitrate N content (NO_3_^−^ -N) (**e**,**f**) under different rice growth stages in 2020 and 2021. CK, Control; F100, 100% CF; MSF100, 100% CF+CMV+RS; MSF80, 80% CF+CMV+RS; MSF60, 60% CF+CMV+RS; MSF40, 40% CF+CMV+RS. CIS, Chinese milk vetch incorporation stage; BTS, before transplanting rice; TS, tillering stage of rice; BS, booting stage of rice; HS, harvesting stage of rice. Asterisks indicate a significant difference at * *p* < 0.05, ** *p* < 0.01, and *** *p* < 0.001.

Applying CMV and RS with reduced CF consistently positively impacted Soil TN content across two growing seasons (Figure 1a,b). The highest soil TN in MSF80 was noted at BS, with an increase of 23.6%, followed by the HS, with an increase of 17.4% over F100. The soil TN was significantly higher with the treatments of CMV and RS with reduced CF at all stages compared with the sole CF treatment in 2021. Compared with F100, the MSF80 increased the soil TN by 18.9% at TS, closely followed by CIS, which increased by 18.0%. The MSF80 at BTS and HS also demonstrated considerable improvements, with increases of 14.9% and 14.4% over F100, respectively.

Results showed that the soil ammonium (NH_4_^+^N) content was significantly affected by the treatments of CMV and RS with reduced CF combination at CIS, TS, and HS in 2020, while in 2021, the CIS, BTS, BS, and HS were recorded significant by the treatments received CMV and RS with reduced CF combination versus sole application of CF (Figure 1c,d). In 2020, compared with F100, the MSF80 increased the NH_4_^+^-N by 57.5% and 64.1% at CIS and TS, respectively. The increased soil NH_4_^+^-N content trend under MSF80 continued in the 2021 growing season. Treatments with CMV and RS with reduced CF significantly affected the NO_3_^−^-N content at TS and HS in 2020, while in 2021, a significant difference was observed at CIS, TS, and BS of rice (Figure 1e,f). Compared with F100, the MSF80 increased soil NO_3_^⁻^- N content by 33.6% and 30.6% at TS of rice in 2020 and 2021, respectively.

**Figure 2:**
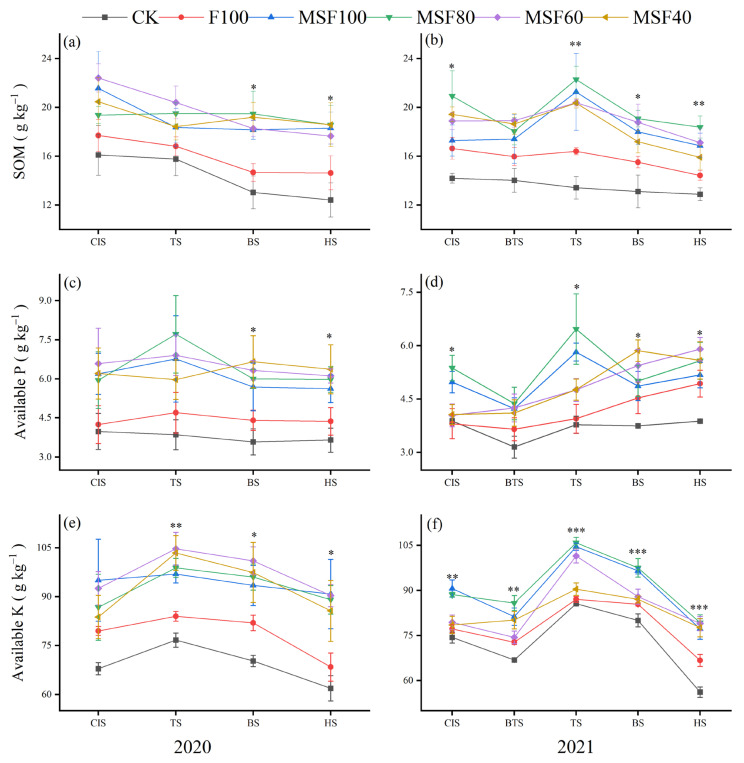
Soil organic matter (SOM) (**a**,**b**), available phosphorus (AP) (**c**,**d**), and available potassium (AK) (**e**,**f**) under different rice growth stages in 2020 and 2021. CK, Control; F100, 100% CF; MSF100, 100% CF+CMV+RS; MSF80, 80% CF+CMV+RS; MSF60, 60% CF+CMV+RS; MSF40, 40% CF+CMV+RS. CIS, Chinese milk vetch incorporation stage; BTS, before transplanting rice; TS, tillering stage of rice; BS, booting stage of rice; HS, harvesting stage of rice. Asterisks indicate a significant difference at * *p* < 0.05, ** *p* < 0.01, and *** *p* < 0.001.

The effect of CMV and RS with reduced CF on SOM in 2020 and 2021 is presented in (Figure 2a,b). The application of CMV and RS with reduced CF significantly affected the SOM at most stages in 2020 and 2021. Compared with the F100 treatment in 2020, the MF80 significantly increased SOM at BS and HS by 32.7% and 26.6%, respectively. In 2021, over F100, the MF80 significantly increased SOM by 18.6%~22.9% at CIS, TS, BS and HS.

The combined application of CMV and RS with reduced CF significantly affected soil available P at most of the stages in both seasons (Figure 2c,d). Plants treated with CMV and RS with reduced CF had higher soil AP than F100. In 2020, replacing 20% of CF with CMV and RS treatment significantly increased the soil AP at BS and HS, which increased by 36.1% and 36.8%, respectively, compared with F100. In 2021, the MSF80 significantly increased the soil AP by 41.3%, 64.1%, 10.5%, and 12.9% at CIS, TS, BS, and HS, respectively, over F100. Soil AK levels were also positively affected by the MSF80 treatment compared to F100 across the seasons (Figure 2e,f). Compared with F100, the MSF80 in 2021 significantly increased the soil AK at all stages by 12.4%~17.5%.

### 2.2. Rice Crop N, P, and K Uptake

**Figure 3:**
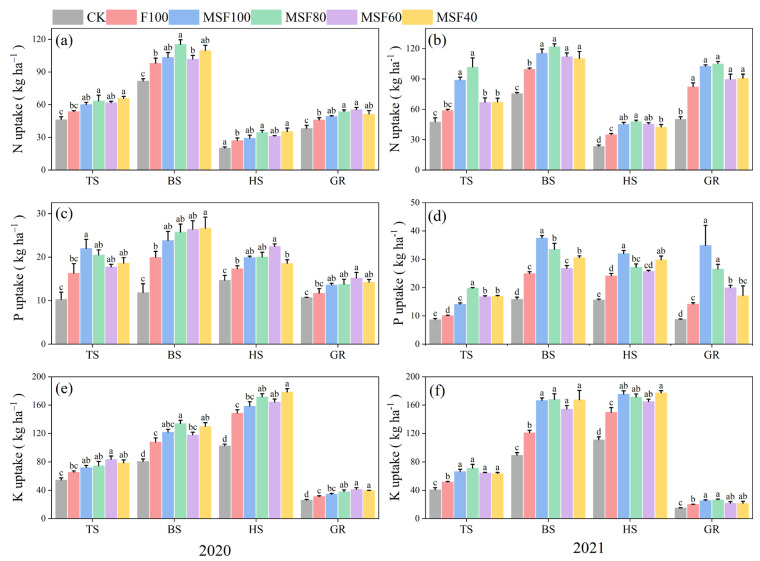
Nitrogen (**a**,**b**), phosphorus (**c**,**d**), and potassium uptakes (**e**,**f**) across different treatments and rice growth stages in 2020 and 2021 seasons. CK, Control; F100, 100% CF; MSF100, 100% CF+CMV+RS; MSF80, 80% CF+CMV+RS; MSF60, 60% CF+CMV+RS; MSF40, 40% CF+CMV+RS. Nutrient uptake in stems and leaves at TS, tillering stage of rice, BS booting stage of rice), and HS harvesting stage of rice). GR means nutrient uptake in rice grain at HS. The different letters above bars indicated statistical differences at *p* < 0.05.

The results showed that CMV and RS with reduced CF significantly affect the N uptake at different growth stages in both years (Figure 3a,b). During 2020, MSF80 increased the N uptake compared with F100, with improvements of 18.2%, 17.6%, and 27.6% at the TS, BS, and HS stages, respectively. The enhancement was more pronounced in 2021, where MSF80 demonstrated remarkable increases of 72.5%, 22.2%, and 36.9% at TS, BS, and HS, respectively. Notably, grain N uptake under MF80 showed substantial improvements of 16.6% and 27.8% over F100 in 2020 and 2021, respectively.

The data demonstrate that treatments received CMV and RS with reduced CF significantly increased P uptake compared with single CF treatment (Figure 3c,d). In 2020, MF80 treatment resulted in significant increases of 25.5%, 28.9%, and 15.3% at TS, BS, and HS stages, respectively, compared with F100. In 2021, compared with F100, MSF80 significantly increased the P uptake by 97.1%, 34.2%, and 12.3% at TS, BS, and HS, respectively. Grain P uptake followed a similar trend, with MF80 demonstrating a significant increase of 17.4% and 87.3% over F100 in 2020 and 2021, respectively.

The treatments that have received the combination of CMV, RS, and CF significantly increased K uptake versus CF alone (Figure 3e,f). During 2020, MF80 treatment significantly increased the K uptake by 13.8%, 23.9%, and 15.5% at TS, BS, and HS stages, respectively, over F100. Though with slightly different magnitudes, the trend continued in 2021 and showed improvements of 38.1%, 38.9%, and 14.4% at the TS, BS, and HS stages, respectively. Grain K uptake under MF80 demonstrated notable increases of 21.8% and 32.2% over F100 in 2020 and 2021, respectively.

### 2.3. Fertilizer Use Efficiency

**Figure 4:**
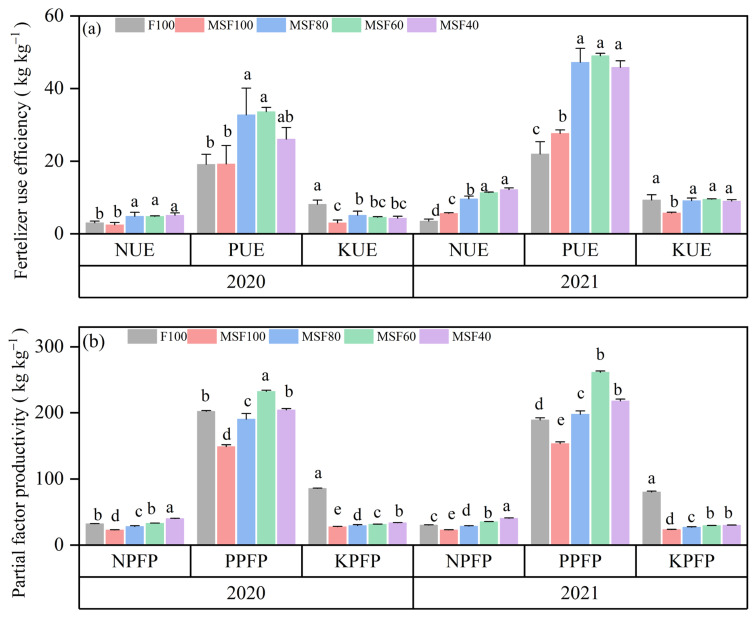
Fertilizer use efficiency (FUE) (**a**) and partial factor productivity (PFP) (**b**) from applied N, P, and K inputs under different treatments in 2020 and 2021 rice seasons; F100, 100% CF; MSF100, 100% CF+CMV+RS; MSF80, 80% CF+CMV+RS; MSF60, 60% CF+CMV+RS; MSF40, 40% CF+CMV+RS. The different letters above bars indicated statistical differences at *p* < 0.05.

The combination of CMV and RS with reduced CF significantly improved nitrogen use efficiency (NUE) in both years of the study (Figure 4a). The MSF40 treatment, which used only 40% CF along with the organic amendments, showed the highest NUE, even though it was not statistically different from the MSF60 treatment. This trend continued for phosphorus efficiency (PUE), with MSF40 being on top again, showing higher efficiency than F100 in 2020 and 2021. Compared with F100, MSF40 significantly increased the NUE and PUE by 40.7% and 32.1%, respectively, in 2020. In 2021, MSF60 significantly increased NUE and PUE by 116.7% and 105.2%, respectively, over F100. However, potassium use efficiency (KUE) was higher in the F100 treatment. The CMV and RS treatments demonstrated greater PFP for N, P, and K than the F100 treatment across the years (Figure 4b). These integrated treatments were more efficient at converting applied nutrients into grain yield. In conclusion, this study makes a strong case for integrating organic amendments (CMV and RS) with reduced CF use.

### 2.4. Grain Yield, Yield Components, and Stability of Rice

**Figure 5:**
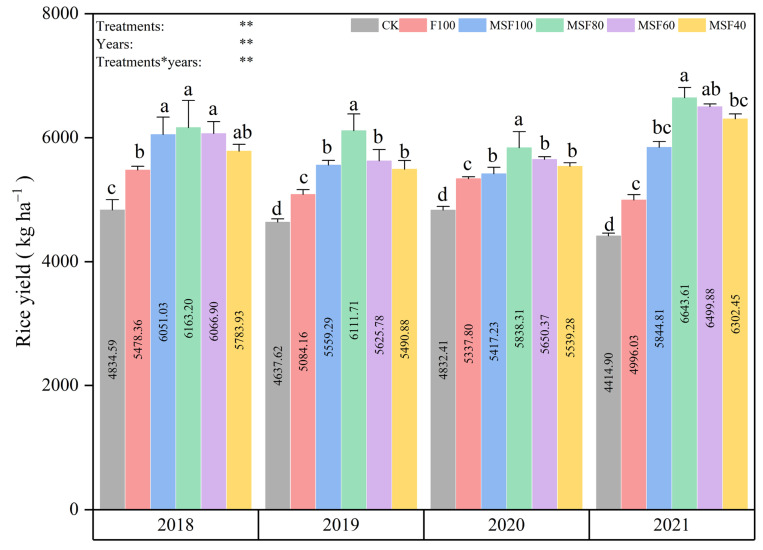
Rice grain yields influenced by various treatments over four years (2018–2021). CK, Control; F100, 100% CF; MSF100, 100% CF+CMV+RS; MSF80, 80% CF+CMV+RS; MSF60, 60% CF+CMV+RS; MSF40, 40% CF+CMV+RS. The different letters above bars indicated statistical differences at *p* < 0.05. Asterisks indicate a significant difference at ** *p* < 0.01.

All fertilizer treatments produced higher annual rice grain yields than CK, suggesting that proper fertilization management can boost rice yields. The results show that CMV and RS received 80% CF treatment had higher yearly grain yields (Figure 5). Compared with the F100 treatment, MF80 significantly increased the rice grain yield in four years by 10.7%, 10.6%, 5.8%, and 24.5%, respectively. There were no statistical differences between the treatment of MF100 and MF40. Compared with the mean yield of the F100 treatment of all years, the MSF80 treatment produced a significantly higher mean grain yield, which increased by 32.2% on average.

The treatments with CMV and RS with reduced CF significantly affected the effective panicle number, grain panicle number, 1000 grain weight and harvest index compared with CK treatment (Table 1). In 2020 and 2021, the highest effective panicle number was noted in MSF40 over F100, which increased by 25.1% and 23.2%, respectively. Compared with F100, the higher grain per panicle, 1000 grain weight, and harvest index were recorded in MSF80 treatment in both years.

**Figure 6:**
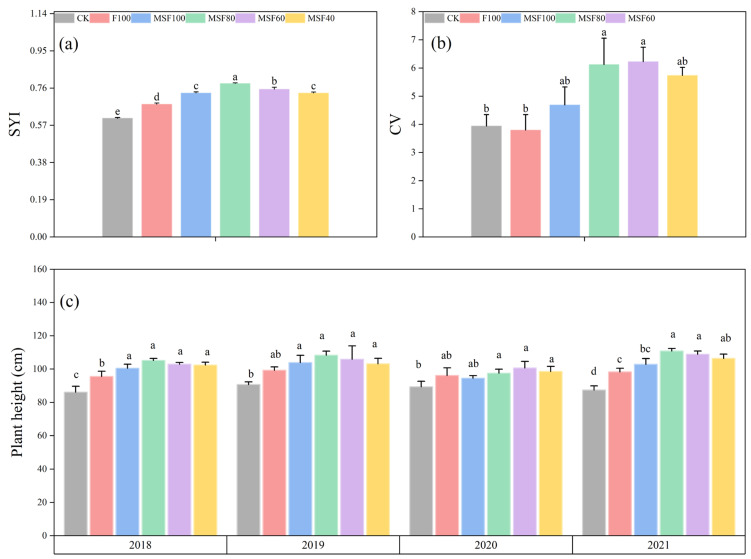
Yield variation coefficient (CV%) (**a**), sustainable yield index (SYI) of rice (**b**) and plant height (**c**) across different treatments at the harvesting stage over four years (2018–2021). CK, Control; F100, 100% CF; MSF100, 100% CF+CMV+RS; MSF80, 80% CF+CMV+RS; MSF60, 60% CF+ CMV+RS; MSF40, 40% CF+CMV+RS. The different letters above bars indicated statistical differences at *p* < 0.05.

The sustainable yield index (SYI) was highest in the MSF80 treatment, whereas the lowest was in the CK treatment (Figure 6a). Plots treated with CMV and RS with different CF rates had a higher SYI than the sole application of CF. Compared with a single application of CF treatment, the highest SYI value was found in the MSF80 treatment, which increased by 23.8%. For yield stability, the highest CV value was observed in the CK treatment (Figure 6b). The CV values dropped when CMV and RS were applied with lower CF treatments, and the incorporation of CMV and RS application increased rice yield stability. Such results imply that CMV substituting CF at appropriate rates (e.g., 20~40%) can potentially maintain sustainable production for rice grain yield under the single-rice cropping system.

Compared with a single application of CF treatment, treatments received CMV and RS with CF significantly increased the plant height (Figure 6c). However, no statistical difference was found between the treatments of MF100, MF80, and MF60 across the years. The highest plant height was recorded in MF80 treatment over four years, which increased by 10.7%, 9%, 1.3%, and 12.1%, respectively, over F100 treatment.

### 2.5. Interactions Between Plant and Soil Properties

**Figure 7:**
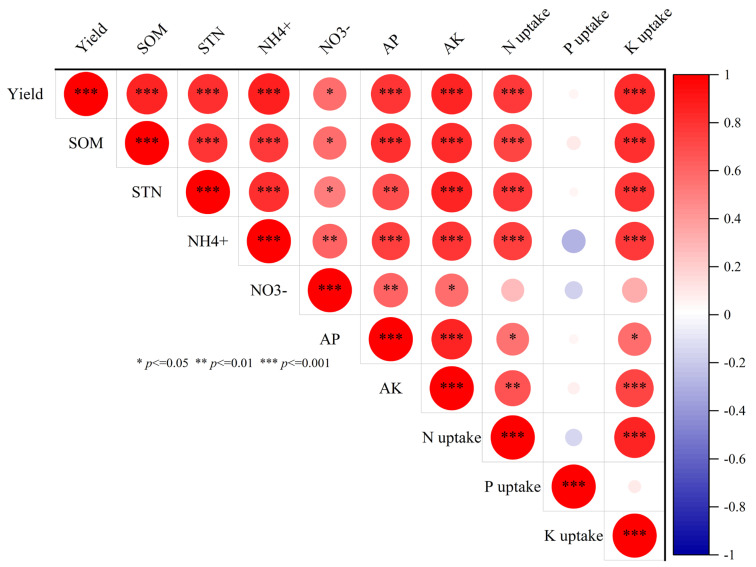
Correlation matrix of soil properties, nutrient uptake, and crop yield parameters. The matrix included parameters such as rice grain yield, SOM (Soil organic matter), STN (soil total N), NH_4_^+^ (Ammonium), NO_3_^−^ (Nitrate), AP (available phosphorus), AK (available potassium), N uptake (Nitrogen uptake), P uptake (Phosphorus uptake), and K uptake (Potassium uptake).

The Pearson correlation analysis showed that yield strongly correlated with several key factors, including SOM, TN, NH_4_^+^, NO_3_^−^, AP, and AK (Figure 7). This suggested that these nutrients play crucial roles in promoting rice productivity. Notably, SOM exhibits strong positive correlations with TN, NH_4_^+^, AP, and AK, highlighting its importance in soil fertility. The different forms of N (TN, NH_4_^+^, and NO_3_^−^) are strongly interrelated and positively associated with yield, emphasizing the significance of N management in rice cultivation.

AP and AK showed strong positive correlations with each other and yield, underscoring their combined importance in rice nutrition. P uptake appears to have weak correlations with most parameters, suggesting it may be less critical for rice yield than other nutrients in this study. In contrast, K uptake showed moderate to strong positive correlations with most parameters. These findings collectively emphasize the complex interplay between SOM, various forms of N uptake, P uptake, and K uptake in influencing rice yield, providing valuable insights for optimizing nutrient management strategies in rice cultivation.

**Figure 8:**
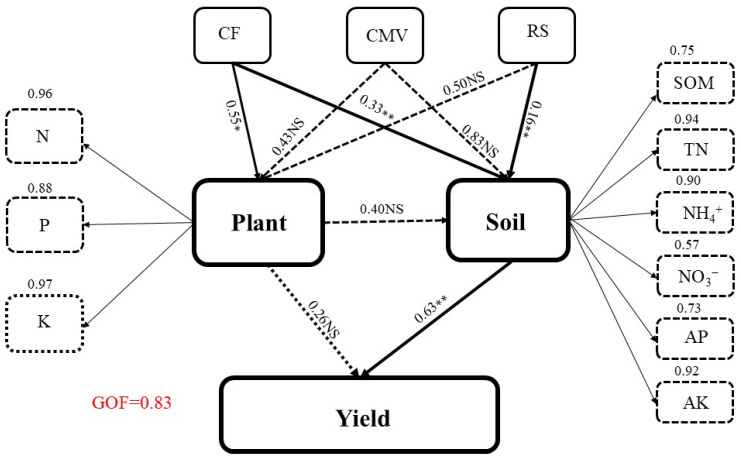
The Partial Least Squares Path Modeling based on the impacts of CMV and RS with CF on nutrient uptake, soil properties, and rice grain yield. Each ellipse represents either the observable or latent variables. The dotted rectangle depicts the loading for soil and plant nutrient uptake, generating the latent variables. The numbers next to the arrows are the standardized path coefficients. Positive and negative effects are indicated by continuous and dashed arrows, respectively. Arrow width is proportional to the strength of path coefficients. Significantly different from zero coefficients are denoted by * *p* < 0.05 and ** *p* < 0.01; NS, not significant. The goodness of fit statistic, which measures overall prediction performance, is used to evaluate the model (goodness of fit = 0.83).

The Partial Least Squares Path Model (PLS-PM) reveal the substantial correlations between CMV and RS application with CF, soil nutrients, nutrient uptake, and rice yield (Figure 8). The use of CMV and RS had a positive effect on CF, suggesting that increased application of CMV and straw leads to greater utilization of fertilizer. Both CMV and RS positively impacted soil nutrients and the absorption of nutrients by rice plants. This phenomenon can be attributed to the synergistic impact of CMV and RS, which enhance the development of rice crops and increase nutrient uptake. Soil nutrients also showed a positive indirect effect on yield via nutrient uptake. The model presented an excellent overall fit (0.83), suggesting that these paths potentially explain the significant relationships between the variables and rice yield.

## 3. Discussion

Assessing rice system sustainability involves considering two key indicators: soil fertility and rice production [21]. The results showed that combining CMV and RS with reduced CF treatments increased the soil TN, mineral N, SOM, AP, and AK at different stages of rice in both 2020 and 2021 compared with F100 (Figure 1 and Figure 2). Applying organic fertilizers and RS to the soil to improve soil fertility and increase soil C and N pools have long been advocated and practiced [22]. External applications of organic materials, such as CMV and RS, can contribute to soil C input directly or indirectly through higher plant biomass, such as crop residues, litterfall, and root systems [23]. In addition, the decomposition of CMV enhanced the NH_4_^+^-N levels and increased the release of N when it returned to the paddy soil. This provided nutrients for rice growth and facilitated N absorption and uptake [3]. It could be the reason for improving TN, NO_3_^−^-N, and SOM in this study (Figure 1 and Figure 2). The soil AP and AK were also increased by combining CMV and RS with CF in different stages (Figure 2). Incorporating GM practices in agroecosystems improves P availability and reduces mineral P-fertilizer input [24]. The combination of CMV and RS has been shown to boost microbial populations in the soil significantly. These microbes play a crucial role in nutrient cycling, particularly in the mineralization of organic P compounds into inorganic forms that are more readily available for plant uptake [25]. Furthermore, the presence of organic acids can enhance the solubility of specific primary minerals that contain P, thereby increasing the overall availability of P [26]. Soil AK content improved significantly during the organic residue release process; for example, soil AK concentrations in CMV decomposition treatment were 134.5% of those observed in blank soil. K in GM exists in plant tissues as K^+^ and is quickly released by water leaching [27]. Rice straw returning increased C and K inputs directly and influenced crop residues, determining the benefits of agricultural SOC sequestration [28].

As expected, plants treated with CMV and RS application accumulated more nutrients than F100 treatment. The results showed that the uptake of nutrients was significantly increased in different stages of rice (Figure 3). Higher uptake in rice with CMV and RS treatment can be attributed to the slow release of nutrients through organic matter decomposition, enhanced soil microbial activity, and improved nutrient retention compared with CF application alone. Adding CMV and RS promotes healthier root systems due to improved soil conditions. A robust root system can explore a larger volume of soil, leading to increased uptake of nutrients. The extensive root networks of leguminous plants also contribute to better nutrient absorption by enhancing the soil’s physical properties [29].

Combining CMV and RS with reduced CF enhanced the FUE and PFP (Figure 4). This was due to the presence of N, P_2_O_5_, and K_2_O in the CMV and RS, which possessed the same nutrients present in CF for rice cultivation. GM, particularly leguminous crops, are rich in N and other nutrients. When incorporated into the soil, they gradually decompose and release these nutrients, providing a steady supply that complements the reduced CF. This gradual release helps maintain nutrient availability over time, allowing rice plants to absorb nutrients more effectively compared to the rapid nutrient release from CF alone [30].

Previous research has shown that growing GM and RS returns significantly impact rice yields [31]. In this study, the results revealed that the effect of organic amendments (CMV+RS) with reduced CF treatment significantly increased rice grain yield compared with sole application CF treatment (Figure 5). The results were consistent with previous research [32] and verified that the application of CMV had a beneficial impact on rice yields. Our findings indicated that combining CMV and CF could supply essential nutrients for rice growth. Furthermore, combining CMV and RS with 80% CF treatment significantly improved the rice grain yield components compared to the F100 treatment (Table 1). This could explain the agronomic process through which the combination of RS and CMV helps maintain or enhance grain yield while reducing the need for synthetic fertilizers. Combining CF and CMV can significantly enhance rice yields by stimulating key components like effective panicle number, gain number per spikelet, and 1000-grain weight. Our results reveal that the treatment with CMV and RS with 80% CF improved SYI and CV values when compared with the F100 treatment (Figure 6), indicating that substituting CF with CMV and RS has a positive effect on the sustainability of annual productivity in the single-rice cropping system [32]. The plant height indicates the vegetative growth potential, a key characteristic that genetic and environmental factors impact. Our findings showed that combining CMV and RS with reduced CF significantly improved plant height (Figure 6), possibly due to the appropriate supply of nutrients. Incorporating RS into the soil can improve its organic matter content, enhancing the soil’s nutrient-holding capacity. The increased organic matter supports better root development and nutrient uptake, leading to taller plants. Studies indicate that organic amendments like RS can significantly improve soil total organic C content, which is crucial for plant growth [25].

The PLS-PM model found that GM and RS application increased rice output by changing soil nutrients, with soil total N being the most significant parameter (Figure 8). Previously, ref. [19] revealed a close correlation of soil mineral N with grain yield and N uptake. The C/N ratios of residues have also been shown to influence soil N availability for succeeding crops. The C/N ratios of residues have also been proven to alter soil N availability for the following crops [33]. Residues with a C/N ratio of less than 25 results in net N mineralization, while those with a greater than 25 result in net N immobilization [34]. Combining legume and non-legume residues may change N mineralization by adjusting C/N ratios. This delays N mineralization and release from legume residues, reducing leaching losses and increasing nutrient availability [35]. The dynamic nutrient uptake observed in our study indicated that rice efficiently capitalized on the delayed release of N. Specifically, the co-incorporation management method resulted in the highest available nutrients in the soil across all growth stages. Furthermore, this approach enhanced the nutrient uptake of rice during both the jointing and maturity phases compared to the application of mineral fertilizers alone.

## 4. Materials and Methods

### 4.1. Experimental Site

**Figure 9:**
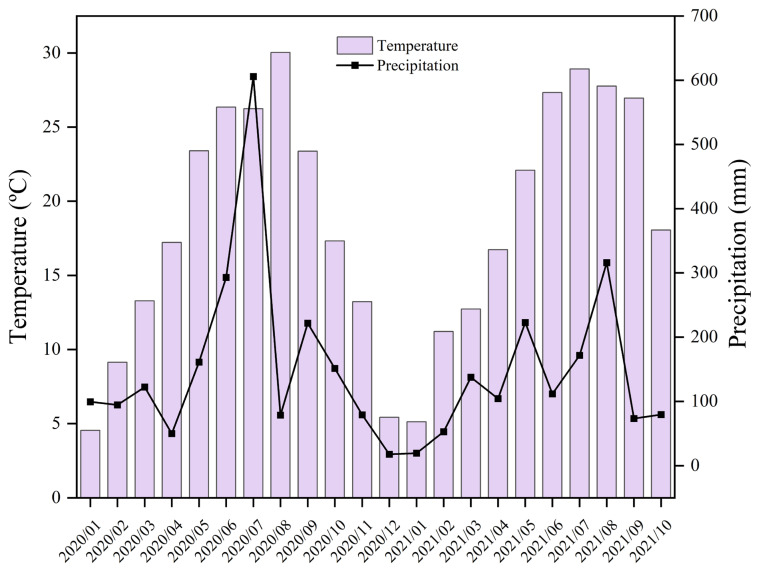
Mean monthly temperature and total monthly precipitation at the experimental site from January 2020 to October 2021.

The present study was set up in 2016 and conducted at an experimental farm of Huazhong Agricultural University in Wuhan City (30°37′ N, 114°21′ E, 27 m above sea level). The texture of the soil was silty clay loam with a yellowish-brown color, having a proportion of 33.8% clay, 53.1% silt, and 13.1% sand. The region under the subtropical zone of the Yangtze River Basin possesses a typical climate with 15 °C to 18 °C mean monthly temperature, ≥1100 mm of mean monthly precipitation (Figure 9), and 230–300 days of frost-free period. The other field operations are shown in Table 2.

### 4.2. Field Management and Experimental Design

A randomized complete block design was followed with three replications of each treatment. Each plot was 30 m^2^ and segregated with 20 cm high and 30 cm wide ridges covered with polyethene to avoid water and nutrient exchange between plots. The field experiment consisted of 6 different treatments, namely CK (Control), F100 (100% recommended CF), MSF100 (100% CF+CMV and RS incorporation), MSF80 (80% CF+CMV+RS), MSF60 (60% CF+CMV+RS), MSF40 (40% CF+CMV+RS). The recommended fertilizer application rate in the rice season is N-P-K: 165, 26.2, 62.3, kg ha^−1^. Urea, superphosphate, and KCL were used as fertilizer sources. Half of the N fertilizer was applied as base fertilizer, 1/4 was applied at the tillering stage, and the remaining was applied at the booting stage. All P fertilizers were applied once before rice transplanting. For K fertilizers, 2/3 was used as a base, and 1/3 was applied at the booting stage. CMV seed (*Yijiang*) was sowed at a 30 kg ha^−1^ seeding rate. The rice cultivar *Huanghuazhan* was used in this study. Rice seedlings were transplanted with 15 × 20 cm^2^ spacing. The total input of N, P, and K from CFs and organic materials for each treatment is shown in Table 3.

### 4.3. Rice Yield and Yield Components

Rice grain yield and plant height were calculated for each treatment from 2018 to 2021. In addition, rice yield components were measured over a 2-year period (2020 and 2021). The crop was harvested manually and then threshed by the thresher. Grain yield was expressed as grams per hill at 14% moisture content, while harvest index (HI) was determined as the ratio of grain yield to total biomass at maturity. Both hills were selected from each pot to obtain the agronomic traits, including plant height, number of spikelets (panicle^−1^), filled grain (%), and 1000-grain weight (g).

### 4.4. Plant and Soil Sample and Analysis

Soil samples were collected in 2020 and 2021 from the 0–20 cm layer at the CMV incorporation (CIS) before rice transplanting (BTS), tilling (TS), booting (BS), and harvesting stages (HS). Five samples were collected randomly through Augur in each plot, pooled and mixed as one sample. The samples were collected in plastic bags after the easily observable crop residues were removed and returned to the laboratory for further analysis. Soil samples were air-dried, crushed, and pulverised to pass through a 1 mm sieve for available P and K analysis. A part of the subsample was crushed further to pass through a 0.25 mm screen for total N and OM analysis. After oxidation with K_2_Cr_2_O_7_, SOM was evaluated using a titration method. Kjeldahl digestion was used to determine the total N [36]. A digital flow analyser was used to analyse soil NO_3_^−^-N and NH_4_^+^-N. The molybdenum blue method was used to determine soil-accessible P (AP) after extracting it with 0.5 M NaHCO_3_ [37]. The AK concentration was determined by flame photometry after extraction with 1 M ammonium acetate using the technique outlined by [38].

Plant samples were collected in both seasons (2020 and 2021) at the TS, BS, and HS. Sub-samples of RS and grains were collected and dried at 60 °C before being weighed and crushed through a 1 mm sieve. A Kjeldahl digestion method was used to determine the N concentration from approximately 0.2 g of ground plant samples [39], the P concentration by molybdovanadate method [40], and the K concentration by flame photometry [38].

### 4.5. Statistical Analysis

The sustainable yield index (SYI) was calculated by comparing the highest observed yield over time under the rice planting technique [41]. A higher SYI number indicates a higher level of sustainability in the system. The SYI was calculated as follows.

$$SYI=\frac{\left(\bar{Y}-\sigma n-1\right)}{Ymax}\times 100$$

$$CV=\frac{\left(\sigma n-1\right)}{\bar{Y}}\times 100$$

$\bar{Y}$ (kg ha^−1^) is the average yield over four years, Y_max_ (kg ha^−1^) is the maximum yield obtained in any year under that treatment, and *σ*_n−1_ is the standard deviation of yield for specific treatment across years.

The fertilizer NUE was defined as yield increase per unit of applied N:

$$Fertilizer\;NUE=\frac{\left(Y-Y_{ctrl}\right)}{F_{n}}$$

The partial factor productivity from applied N (NPFP) was the grain yield produced by applied N as

$$NPFP=\frac{\left(Y\right)}{F_{n}}\times 100$$

Y and Y_ctrl_ are the total grain yields in 2021 with and without N application. F_n_ is the total exogenous N applied, comprising inorganic fertilizer application and the N generated from biological fixation. The natural ^15^N abundance approach was used to calculate the fraction of biological N fixation to total N in CMV plants (74.4% on average), according to [42]. The calculation of fertilizer P and K use efficiency, and PPFP and KPFP were analogous to N.

Harvest index (%) = rice yield/(rice yield + RS yield) × one hundred

Nutrient uptake (kg ha^−1^) = dry matter mass (kg ha^−1^) × Nutrient content (%)

One-way ANOVA tests were used to compare the results of different treatments at the *p* < 0.05 level. Duncan’s multiple range test was used to separate means on significant ANOVA testing using SPSS 20.0. The R package ‘plspm’ version 0.5.0 was used for the PLS-PM.

## 5. Conclusions

Following four years of continuous CMV and RS treatment combined with reduced CF, soil NO_3_^−^-N, TN, SOM, AK, and AP contents were improved compared with CK and F100 treatment. The study also observed marked improvements in nutrient uptake and fertilizer use efficiency, particularly for N and P, demonstrating the potential for substantial reductions in fertilizer inputs without compromising productivity. In addition, treatments with CMV and RS combined with 20% of the recommended dose of CF produced higher rice yields than the single CF and improved the yield components and the SYI. Therefore, CMV and RS incorporation with 20% CF reduction can be an effective fertilization management strategy for the sustainable production of rice.

## Figures and Tables

**Table 1:** Effect of Chinese milk vetch and rice straw with the combination of chemical fertilizer on yield components.

Year	Treatment	Effective Panicle Number (×104 ha^−1^)	Number of Grains per Panicle	Seed Setting Rate (%)	1000-Grain Weight (g)	Harvest Index (%)
2020	CK	192.81 ± 2.04 d	97.27 ± 0.88 b	53.35 ± 0.25 a	16.63 ± 0.23 b	40.39 ± 0.28 b
	F100	215.06 ± 3.35 c	101.04 ± 3.20 b	55.04 ± 0.55 a	17.51 ± 0.49 b	44.88 ± 0.36 a
	MSF100	246.98 ± 3.13 b	107.91 ± 2.42 a	57.89 ± 1.99 a	18.16 ± 0.78 ab	46.00 ± 1.09 a
	MSF80	268.02 ± 3.47 a	112.14 ± 1.04 a	61.68 ± 2.97 a	19.54 ± 0.41 a	47.78 ± 1.45 a
	MSF60	253.32 ± 4.01 b	111.01 ± 1.11 a	59.50 ± 0.71 a	18.26 ± 0.33 ab	47.28 ± 2.46 a
	MSF40	269.33 ± 5.65 a	107.51 ± 2.40 a	61.25 ± 3.17 a	18.03 ± 0.54 ab	45.08 ± 0.82 a
2021	CK	210.71 ± 2.68 d	111.78 ± 0.88 c	51.88 ± 0.33 a	18.84 ± 0.33 b	41.01 ± 0.75 b
	F100	232.63 ± 4.32 c	115.55 ± 3.20 bc	53.57 ± 0.54 a	18.96 ± 0.18 b	43.45 ± 1.22 b
	MSF100	264.54 ± 4.41 b	122.42 ± 2.42 ab	55.42 ± 1.99 a	19.44 ± 0.69 b	46.53 ± 1.03 a
	MSF80	286.92 ± 3.47 a	127.65 ± 1.37 a	59.21 ± 2.97 a	19.45 ± 0.15 b	47.17 ± 0.85 a
	MSF60	275.56 ± 12.37 ab	127.18 ± 1.75 a	58.36 ± 0.94 a	20.75 ± 0.22 a	48.01 ± 0.87 a
	MSF40	286.23 ± 7.33 a	122.02 ± 2.40 ab	58.78 ± 3.17 a	19.63 ± 0.09 b	47.09 ± 0.73 a

Note: Data in a column followed by various letters are significant among treatments at *p* < 0.05.

**Table 2:** Dates of field operations during the study period.

Field Operations	Date	
	2019–2020	2020–2021
Sowing of Chinese milk vetch	22 October 2019	13 October 2020
Chinese milk vetch incorporation	17 April 2020	9 April 2020
Basal fertilizer application	13 June 2020	18 June 2021
Rice transplantation	14 June 2020	20 June 2021
Fertilizer topdressing at the tillering stage	24 June 2020	29 June 2021
Fertilizer topdressing at the booting stage	6 August 2020	15 August 2021
Rice harvesting	12 September 2020	21 September 2021

**Table 3:** Total input of nitrogen (N), phosphorus (P), and potassium (K) for different treatments in 2020 and 2021 seasons.

Treatment	NPK Input via Inorganic Fertilizer			NPK Input via CMV			NPK Input via RS			Total (kg ha^−1^)		
	N	P	K	N	P	K	N	P	K	N	P	K
CK												
F100	165.0	26.4	62.3							165.0	26.4	62.3
MSF100	165.0	26.4	62.3	31.4	4.6	33.0	41.0	19.9	99.1	237.3	50.9	194.3
MSF80	131.9	21.1	49.8	32.3	4.0	41.4	43.2	20.0	105.7	207.4	45.1	196.9
MSF60	98.9	15.8	37.4	28.6	3.3	39.1	43.3	22.4	102.7	170.8	41.5	179.2
MSF40	65.0	17.6	24.9	30.8	4.3	39.0	41.9	18.5	99.8	137.8	40.4	163.8
CK	-	-	-							N	P	K
F100	165.0	26.4	62.25							165.0	26.4	62.3
MSF100	165.0	26.4	62.25	33.0	5.4	39.6	56.6	19.9	147.4	254.6	51.7	249.3
MSF80	131.9	21.1	49.8	40.5	6.1	49.9	59.2	20.0	145.3	231.6	47.2	245.0
MSF60	98.9	15.8	37.4	33.2	4.3	46.6	51.5	22.4	136.0	183.6	42.5	220.0
MSF40	65.0	17.6	24.9	37.1	5.1	47.4	53.0	18.5	137.3	155.1	41.2	209.6

## Data Availability

Data will be made available upon request.
